# Comparative long-term outcomes of conventional immunomodulatory therapy, biologic agents, and combination therapy in chronic uveitis

**DOI:** 10.1186/s12348-026-00591-0

**Published:** 2026-05-08

**Authors:** Charles Zhang, Matthew McSoley, Sinan Ersan, Steven Yeh, Thomas A. Albini

**Affiliations:** 1 Department of Ophthalmology, Bascom Palmer Eye Institute, University of Miami Miller School of Medicine, 900 NW 17th Street, Miami, FL 33136 USA; 2 Department of Ophthalmology, Ross Eye Institute, SUNY Buffalo, Buffalo, NY USA; 3 Jacobs School of Medicine and Biomedical Sciences, University at Buffalo, Buffalo, NY USA; 4 Univeristy of Nebraska Medical Center, Omaha, NE USA

**Keywords:** Chronic uveitis, Immunomodulatory therapy, Methotrexate, Adalimumab, Infliximab

## Abstract

**Background:**

Chronic non-infectious uveitis can lead to vision loss if not adequately controlled. Thus, steroid-sparing immunomodulatory therapy (IMT) is often required to achieve and maintain disease quiescence. It is unclear if biologic agents inhibiting tumor necrosis factor alpha (TNF-α) provide superior control of chronic uveitis compared to conventional IMTs on their own or as combination therapy.

**Methods:**

This is a multicenter retrospective cohort study using the TriNetX US Collaborate network. Adult patients (≥18 years) with chronic non-infectious uveitis who achieved steroid-sparing quiescence on IMT were included. Patients were grouped by initial maintenance with conventional IMT (methotrexate, mycophenolate mofetil, or azathioprine), biologic TNF-α inhibitor (adalimumab or infliximab) or combination therapy (both conventional IMT and biologic agent), and the incidence of flares over 6, 12, 18 and 24 months of follow-up were analyzed. Outcomes were compared between groups using paired chi-squared tests on propensity score matched cohorts with relative risk (RR) and 95% confidence intervals calculated for each outcome.

**Results:**

A total of 2,912 patients on conventional IMT, 736 patients on biologic therapy and 1,313 on combination therapy met the definition of steroid-sparing control. Patients on combination therapy had significantly lower rates of flares as compared to those on conventional IMT at 6 months (RR 0.53; *p* = 0.0070), 12 months (RR 0.52; *p* = 0.0011), 18 months (RR 0.56; *p* = 0.0022) and 24 months (RR 0.56; *p* = 0.0012). No difference in the rate of flares was found between combination versus biologic therapy, or conventional IMT versus biologic therapy at 6-, 12-, 18- and 24 months. Patients initially treated with conventional IMT had higher rates of augmentation or switching to biologic therapy at 12 months (RR 1.74; *p* = 0.0386), 18 months (RR 1.65; *p* = 0.0219) and 24 months (RR 1.65; *p* = 0.0114).

**Conclusion:**

The current study indicates that for patients with chronic uveitis who achieve steroid-sparing control on IMT, those treated with a combination of a conventional IMT and a biologic TNF-α inhibitor have a lower risk of disease activation as compared to those on conventional IMT.

**Supplementary information:**

The online version contains supplementary material available at https://doi.org/10.1186/s12348-026-00591-0.

## Introduction

Chronic non-infectious uveitis can lead to irreversible vision loss and is responsible for 10% of blindness in working age adults in the United States [1, 2]. Each inflammatory flare can lead to cumulative damage to the eye, increasing the risk of glaucoma, macular edema and photoreceptor degeneration [1–3]. Corticosteroids are the cornerstone for initial management of flares; however, long-term steroid use is limited by numerous adverse effects [4]. As such, chronic uveitis often requires additional immunomodulatory therapy (IMT) to maintain remission [4].

Conventional IMTs, including azathioprine, methotrexate and mycophenolate mofetil, are widely used as first-line steroid-sparing agents [5]. Compared with other treatment modalities, these agents remain attractive options due to their lower cost and well-established safety profiles [6, 7]. In contrast, biologic therapies inhibiting tumor necrosis factor- alpha (TNF-α) have emerged as a treatment option for refractory cases of chronic uveitis. In particular, adalimumab became the first FDA-approved systemic agent for non-infectious uveitis after the pivotal VISUAL I and II trials [8, 9]. Although these trials established biologic agents as an effective therapy for uveitis, they are substantially more expensive than conventional IMT. A cost-effectiveness analysis from the United Kingdom’s National Health Service demonstrated considerable financial burden associated with adalimumab, with a higher cost per quality-adjusted life year when used as maintenance therapy for inactive uveitis [10, 11]. As a result, the use of biologic agents has often been reserved for cases that are refractory to conventional IMTs or for patients who cannot tolerate their adverse effects.

However, recently the ADVISE trial demonstrated that, over a 12-month period, adalimumab was more effective than conventional IMT in achieving corticosteroid-sparing disease control in non-infectious intermediate, posterior, or panuveitis [12]. Nonetheless, the comparative long-term efficacy of conventional IMTs, biologic agents and combination therapies for maintenance of disease control remains unclear. The current study aims to address this gap by comparing long-term rates of flare among patients with chronic uveitis who achieved steroid-sparing disease control and were managed with conventional IMT, biologic agents, or combination therapy.

## Methods

### Study design

This is a retrospective cohort study utilizing the TriNetX US Collaborative Network, a federated network that aggregates de-identified electronic health record data from dozens of healthcare organizations in the United States. The network includes a large, geographically diverse patient population in real time from both academic and private institutions. All queries were performed on August 27, 2025, and included data from January 1^st^, 2006 through December 31^st^, 2024. The study was deemed exempt from institutional review board review as only de-identified, aggregated patient data was used. This research adhered to the tenets of the Declaration of Helsinki.

Diagnoses are represented in TriNetX using International Classification of Disease, Tenth Revision, Clinical Modification code set (ICD-10-CM) and RxNorm, a standardized, normalized naming system for clinical drugs in the US. Full details of coding data are provided in supplemental Table 1.

### Study population

All adult patients (≥18 years) diagnosed with chronic non-infectious uveitis who achieved disease control on immunomodulatory therapy were included. To define chronic uveitis in the TriNetX database, patients were required to have a diagnosis code for uveitis on at least two separate encounters > 3 months apart as previously described [13]. Uveitis diagnoses were identified using ICD-10-CM codes for non-infectious intraocular inflammation, specifically anterior uveitis/iridocyclitis (H20.X), intermediate uveitis (H30.2X, coded within chorioretinal inflammation H30.X), and posterior or panuveitis (H30.X, H44.11). This operational definition is consistent with the Standardization of Uveitis Nomenclature (SUN) criteria for chronicity (persistent or recurrent inflammation ≥ 3 months in duration) [14]. Subgroup analyses by specific uveitis diagnosis were not performed because the number of patients within each category was toosmall. To protect patient privacy, analyses involving 10 or fewer patients are suppressed and not reported in the analytics system, which precluded reporting results for individual diagnostic subgroups. Patients were then required to meet a stringent definition of control on steroid-sparing therapy after their uveitis diagnosis. In clinical practice, quiescent uveitis is defined by inactive inflammation with ≤7.5 mg/day of prednisone and less than 2 times daily equivalent dosing of topical prednisolone acetate 1% [7, 12]. Given the limitations of TriNetX database, patients had to have no recorded prescription for oral prednisone ≥ 5 mg (RxNorm code 8640 for 5, 10, 20 or 50 mg tablets) and no active ophthalmic corticosteroid drop prescriptions (prednisolone acetate 1% [RxNorm 1376336], prednisolone sodium phosphate 1% [RxNorm 314165], or difluprednate 0.05% [RxNorm 23043]) for at least 3 months. Patients who met this steroid-sparing criterion while on IMT were considered to have achieved controlled quiescent uveitis for the purposes of analysis.

Eligible patients were categorized into three cohorts based on their immunomodulatory therapy at the time steroid-sparing control was achieved. The conventional IMT cohort consisted of patients who were started on methotrexate (RxNorm 6851), mycophenolate mofetil (RxNorm 68149, excluding topical formulations) or azathioprine (RxNorm 1256) without a history of use of infliximab (RxNorm 191831) or adalimumab (RxNorm 326361) prior to the initiation of the antimetabolite agent. The biologic therapy cohort consisted of patients who were started on infliximab or adalimumab, without a prior history of methotrexate, mycophenolate mofetil or azathioprine. Finally, a combination therapy cohort was defined, comprising patients who were treated with a combination of one of the conventional IMT plus one of the TNF-α inhibitor biologics concurrently. For all groups, the initiation of the immunomodulatory treatment had to occur after the initial uveitis diagnosis code, to ensure that the therapy was indicated for uveitis rather than a pre-existing condition.

To reduce confounding and increase the likelihood that the indication for systemic immunotherapy was uveitis rather than another condition, patients with a history of major autoimmune conditions that commonly necessitate immunosuppressive therapy were excluded. These included any diagnoses of inflammatory bowel disease (Crohn’s disease [ICD-10 K50] or ulcerative colitis [K51]), psoriasis (L40), systemic lupus erythematosus (L93), rheumatoid arthritis (M05–M06), juvenile idiopathic arthritis (M08), ankylosing spondylitis (M45), or other inflammatory spondyloarthropathies (M46).

### Outcomes

The primary outcome of interest was the occurrence of a uveitis flare during follow-up. Because clinical examination data, such as anterior chamber cell grades, were not available in the database, flares were defined indirectly by changes in the management of uveitis. A flare was considered to have occurred if a patient required an increase in anti-inflammatory therapy, specifically a new prescription for oral prednisone at a dose of 10 mg/day or higher. In routine practice, the threshold for corticosteroid-sparing disease control is defined as inactive inflammation on ≤7.5 mg/day of oral prednisone [7, 12]. This threshold was required at cohort entry; therefore, initiation of a new course of ≥10 mg/day of oral prednisone during follow-up was considered indicative of escalation of therapy for a flare of uveitis. Because the TriNetX database does not capture dosing frequency of topical medications, flares managed exclusively with topical prednisolone at > 2 drops/day were not captured. As a result, the flares captured in this study most likely represent moderate-to-severe relapses or those with greater posterior segment involvement. Flare events were assessed cumulatively over time, and the proportion of patients in each cohort who experienced a flare within 6, 12, 18, and 24 months after achieving steroid-sparing control was calculated. The index date for follow-up was set as the first date when the steroid-sparing control criteria were met.

The secondary outcome was treatment “escalation” or switching to the alternate class of immunotherapy. For patients in the conventional IMT group, individuals were tracked whether they later received a prescription for a biologic agent during the follow-up period, which would indicate that a biologic was added to their regimen or that they were switched to a biologic due to inadequate control or intolerance. Due to limitations within the TriNetX framework, these two outcomes could not be differentiated. Conversely, for patients in the initial biologic group, the rate of a prescription of a conventional IMT was evaluated, indicating addition of or switch to an antimetabolite. These events will be referred to collectively as “augmentation” of therapy with the other drug class. Similar to flares, the cumulative rates of these events at 6, 12, 18, and 24 months were calculated.

### Statistical analysis

The TriNetX built-in analytics platform was used to perform propensity score matching (PSM) and create comparable cohorts for outcome comparisons. The propensity model included baseline characteristics such as age, sex, race, ethnicity, and smoking status, identified using ICD-10 codes for nicotine dependence (F17.2x), tobacco use (Z72.0), or personal history of tobacco use (Z87.891), given evidencesuggesting that smoking affects the risk of uveitis flares [15]. Matching was performed between each pair of directly compared treatment groups. This was first performed in conventional and biologic monotherapy groups, followed by comparisons of the combination therapy group and the conventional group, and, separately, with the biologic group. A greedy nearest-neighbor algorithm was used with a caliper of 0.25 standard deviations of the propensity score, and matching was without replacement. Balance of covariates after matching was assessed by p-values and standardized differences.

## Results

### Baseline Characteristics

Before matching, inclusion criteria of patients with chronic uveitis that achieved steroid-sparing control were met by 2,912 patients controlled initially on conventional IMT and 736 patients controlled on biologic agents. In addition, 1,313 patients achieved steroid-sparing quiescence with a combination of conventional IMT and biologic agents. Summary of populations before and after PSM are seen in Tables 1, 2, and 3.

**Table 1:** Baseline characteristics of patients receiving conventional immunomodulatory therapy compared to biologic therapy

Characteristics	Before Propensity Score Matching			After Propensity Score Matching		
	Conventional	Biologic	p-value	Conventional	Biologic	p-value
Total Patients	2912	736	NA	732	732	NA
**Age (Years)**	46.1 ± 20.1	40.3 ± 17.6	<0.0001	40.6 ± 18.5	40.4 ± 17.6	0.8226
**Sex**						
Male	1045	241	0.0895	238	241	0.8673
Female	1826	483	0.1991	486	483	0.8684
Unknown	24	12	0.0501	10	10	1.0000
**Race**						
White	1661	429	0.6544	446	429	0.3649
African American and/or Black	594	152	0.9359	153	152	0.9487
Asian	111	29	0.8939	26	29	0.6801
American Indian or Alaska Native	19	10	0.0559	10	10	1.0000
Native Hawaiian or Other Pacific Islander	16	10	0.0206	10	10	1.0000
Unknown	299	75	0.9125	72	71	0.9298
Other	195	41	0.2523	30	41	0.1808
**Ethnicity**						
Not Hispanic or Latino	2065	506	0.1693	528	506	0.2068
Hispanic or Latino	291	76	0.8255	60	76	0.1497
Unknown Ethnicity	539	154	0.1552	144	150	0.6955
**Social History**						
Tobacco Use	50	10	0.4839	10	10	1.0000
Nicotine Dependence	192	42	0.294	31	39	0.3271
Tobacco Use History	230	53	0.5016	52	53	0.9193

**Table 2:** Baseline characteristics of patients receiving combination therapy compared to conventional immunomodulatory therapy

Characteristics	Before Propensity Score Matching			After Propensity Score Matching		
	Combination	Conventional	p-value	Combination	Conventional	p-value
Total Patients	1313	2912	NA	1298	1298	NA
**Age (Years)**	37.0 ± 20.3	46.1 ± 20.1	<0.0001	37.2 ± 20.3	37.3 ± 20	0.9217
**Sex**						
Male	483	1045	0.654	478	471	0.7754
Female	819	1826	0.6858	810	818	0.7454
Unknown	10	24	0.8226	10	10	1.0000
**Race**						
White	745	1661	0.7196	740	777	0.1406
African American and/or Black	239	594	0.0827	238	228	0.6091
Asian	42	111	0.3096	42	26	0.0493
American Indian or Alaska Native	16	19	0.0624	12	12	1.0000
Native Hawaiian or Other Pacific Islander	10	16	0.4218	10	10	1.0000
Unknown	143	299	0.5756	141	139	0.8993
Other	123	195	0.0027	121	112	0.5366
**Ethnicity**						
Not Hispanic or Latino	880	2065	0.0053	868	894	0.2745
Hispanic or Latino	187	291	<0.0001	186	173	0.4598
Unknown Ethnicity	245	539	0.9659	244	231	0.5093
**Social History**						
Tobacco Use	18	50	0.3974	18	11	0.1911
Nicotine Dependence	79	192	0.4547	79	68	0.3503
Tobacco Use History	90	230	0.3974	90	79	0.3815

**Table 3:** Baseline characteristics of patients receiving combination therapy compared to biologic therapy

Characteristics	Before Propensity Score Matching			After Propensity Score Matching		
	Combination	Biologic	p-value	Combination	Biologic	p-value
Total Patients	1313	736	NA	733	733	NA
**Age (Y, mean ± SD)**	37 ± 20.3	40.3 ± 17.6	0.0002	40.2 ± 19.2	40.3 ± 17.7	0.9358
**Sex**						
Male	483	241	0.0645	223	241	0.3121
Female	819	483	0.1486	504	483	0.2422
Unknown	10	12	0.0674	10	10	1.0000
**Race**						
White	745	429	0.5089	463	429	0.0689
African American and/or Black	239	152	0.1784	141	152	0.4725
Asian	42	29	0.3804	26	29	0.6801
American Indian or Alaska Native	16	10	0.7872	10	10	1.0000
Native Hawaiian or Other Pacific Islander	10	10	0.1878	0	10	0.0015
Unknown Race	143	75	0.6176	63	72	0.4163
Other Race	123	41	0.0023	34	41	0.4067
**Ethnicity**						
Not Hispanic or Latino	880	506	0.4363	522	506	0.3613
Hispanic or Latino	187	76	0.0108	71	76	0.6637
Unknown Ethnicity	245	154	0.2173	140	151	0.4714
**Social History**						
Tobacco Use	18	10	0.9802	10	10	1
Nicotine Dependence	79	42	0.6769	44	41	0.7374
Tobacco Use History	90	53	0.7712	53	52	0.9193

Baseline Characteristics of each of the three cohort groups before and after Propensity Score Matching (PSM). Baselines for the conventional versus biologic, combination versus conventional, and combination versus biologic cohorts are displayed in Tables 1, 2, and 3, respectively.

### Risk of a flares

The cumulative rate of flares were measured in patients with chronic uveitis achieving steroid-sparing control treated initially with conventional IMT and compared to matched patients treated initially on biologic agents over the 6, 12, 18 and 24-month follow-up periods (Table 4). Patients initially treated with conventional IMT had no significant difference in the rate of flares at 6 months (Relative risk [RR] 1.44; 95% confidence interval [CI] 0.77–2.70; *p* = 0.2559), 12 months (RR 1.50; 95% CI 0.86–2.62; *p* = 0.2559), 18 months (RR 1.42; 95% CI 0.87–2.33; *p* = 0.1566) or 24 months (RR 1.55; 95% CI 0.98–2.45; *p* = 0.0563).

**Table 4:** Risk of uveitis flares in patients treated with conventional versus biologic therapy

Time Period	Conventional	Biologic	Risk Ratio	95% CI	*p*-value
6 months	23 (3.1%)	16 (2.2%)	1.44	0.77–2.70	0.2559
12 months	30 (4.1%)	20 (2.7%)	1.50	0.86–2.62	0.1502
18 months	37 (5.1%)	26 (3.5%)	1.42	0.87–2.33	0.1566
24 months	45 (6.1%)	29 (4.0%)	1.55	0.98–2.45	0.0563

Risk of uveitis flare, as defined by a prescription of ≥10 mg of oral prednisone, in patients with chronic uveitis treated with conventional immunomodulatory therapy compared to biologic agents. After propensity score matching, 732 patients were in analyzed in each cohort

**Fig. 1:**
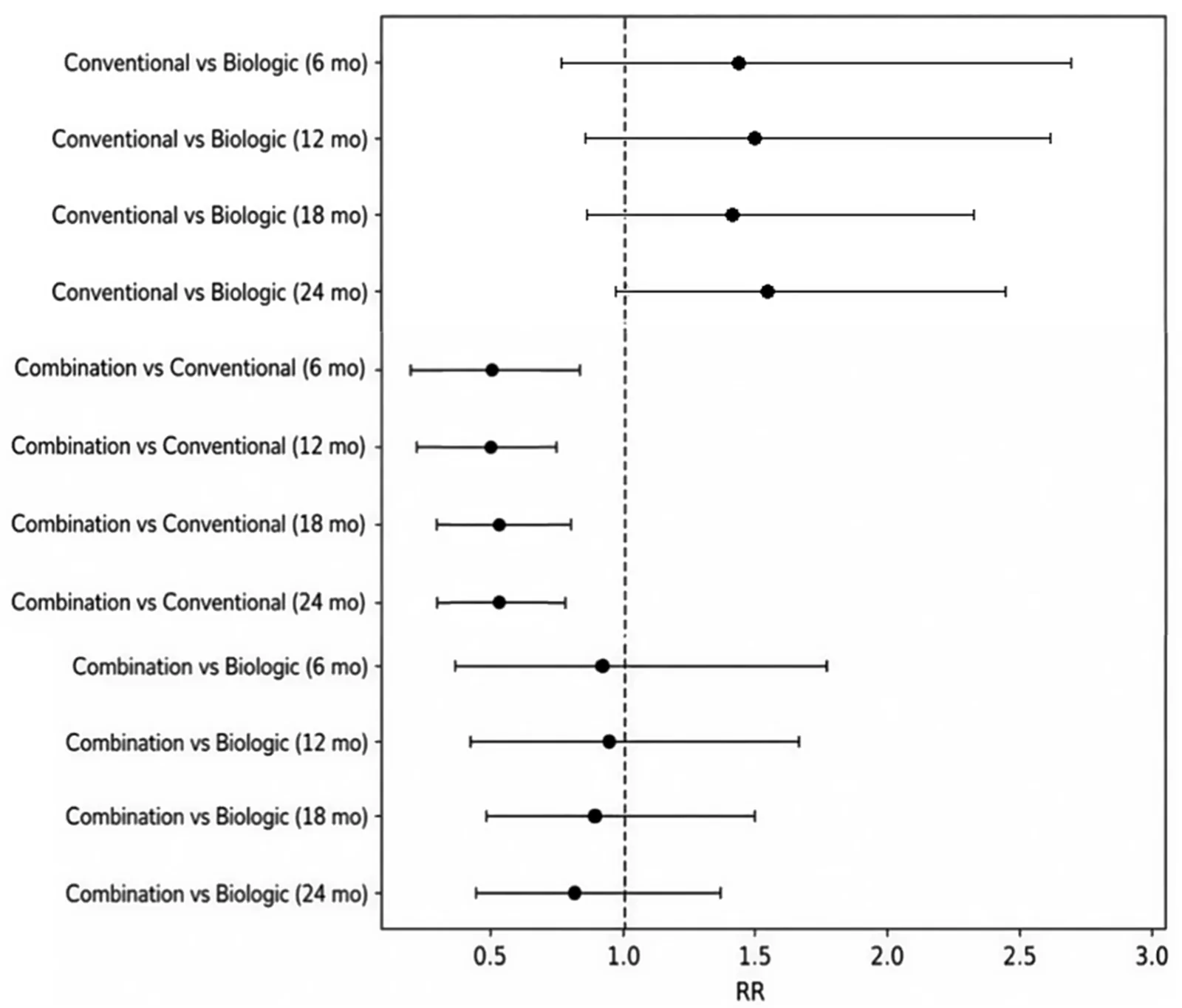
Forest plot illustrating the comparative risk of uveitis flares in patients treated with one of three treatment modalities: conventional immunomodulatory therapy (methotrexate, mycophenolate mofetil or azathioprine), biologic therapy (infliximab or adalimumab) or a combination of the two. All comparisons were analyzed at 6 months, 12 months, 18 months and 24 months after initiation of therapy. This figure was created using ChatGPT (OpenAI).

The cumulative rates of flares were also measured and in patients with combination therapy (conventional IMT and biologic agents) and compared to patients treated initially on conventional IMT alone or biologic agents alone. Combination therapy had significantly lower rates of flares as compared to conventional IMT at 6 months (RR 0.53; 95% CI 0.33–0.85; *p* = 0.0070), 12 months (RR 0.52; 95% CI 0.34–0.77; *p* = 0.0011), 18 months (RR 0.56; 95% CI 0.39–0.82; *p* = 0.0022) and 24 months (RR 0.56; 95% CI 0.39–0.80; *p* = 0.0012) (Table 5). However, combination therapy was found to have no significant difference in the rate of flares as compared to biologic therapy alone at 6 months (RR 0.94; 95% CI 0.47–1.88; *p* = 0.8559), 12 months (RR 0.95; 95% CI 0.51–1.77; *p* = 0.8711), 18 months (RR 0.92; 95% CI 0.54–1.59; *p* = 0.7735) or 24 months (RR 0.86; 95% CI 0.51–1.46; *p* = 0.5791) (Table 6). These ratios as summarized graphically in Fig. 1.

**Table 5:** Risk of uveitis flares in patients treated with combination versus conventional therapy

Time Period	Combination	Conventional	Risk Ratio	95% CI	*p*-value
6 months	26 (2.0%)	49 (3.8%)	0.53	0.33–0.85	0.0070
12 months	34 (2.6%)	66 (5.1%)	0.52	0.34–0.77	0.0011
18 months	41 (3.2%)	73 (5.6%)	0.56	0.39–0.82	0.0022
24 months	44 (3.4%)	79 (6.1%)	0.56	0.39–0.80	0.0012

Risk of uveitis flare, as defined by a prescription of ≥10 mg of oral prednisone, in patients with chronic uveitis treated with combination therapy (conventional immunomodulatory therapy and biologic agents) compared to conventional immunomodulatory therapy alone. After propensity score matching, 1298 patients were analyzed in each cohort

**Table 6:** Risk of uveitis flares in patients treated with combination versus biologic therapy

Time Period	Combination	Biologic	Risk Ratio	95% CI	P
6 months	15 (2.0%)	16 (2.2%)	0.94	0.47–1.88	0.8559
12 months	19 (2.6%)	20 (2.7%)	0.95	0.51–1.77	0.8711
18 months	24 (3.3%)	26 (3.5%)	0.92	0.54–1.59	0.7735
24 months	25 (3.4%)	29 (4.0%)	0.86	0.51–1.46	0.5791

Risk of uveitis flare, as defined by a prescription of ≥10 mg of oral prednisone, in patients with chronic uveitis treated with combination therapy (conventional immunomodulatory therapy and biologic agents) compared to biologic agents alone. After propensity score matching, 733 patients were analyzed in each

### Risk of augmentation of IMT

Next, the frequency with which patients required augmentation or escalation of their therapy with the alternative drug class, as an indirect measure of inadequate disease control or drug intolerance was measured at the 6, 12, 18 and 24-month follow-up periods (Table 7). Importantly, this PSM dataset was the same as the one used to derive the rate of flares in Table 4. No significant difference in the rate of augmentation between patients on conventional IMT was as compared to those on biologic therapy found at 6 months (RR 1.58; 95% CI 0.90–2.78; *p* = 0.1455). However, patients with conventional IMT had significantly higher rates of augmentation at 12 months (RR 1.74; 95% CI 1.05–2.88; *p* = 0.0386), 18 months (RR 1.65; 95% CI 1.09–2.49; *p* = 0.0219) and 24 months (RR 1.65; 95% CI 1.13–2.41; *p* = 0.0114). At each time point in both groups, the number of individuals meeting augmentation criteria was greater than the number of individuals that had a uveitis flare; at the 24-month interval this was 66 (9.0%) requiring augmentation and 45 (6.1%) having a flare in the conventional IMT group and 40 (5.5%) requiring augmentation and 29 (4.0%) having a flare in the biologic therapy group.

**Table 7:** Risk of conversion or supplementation of an alternative class of medication in patient treated with conventional versus biologic therapy

Time Period	Conventional	Biologic	Risk Ratio	95% CI	P
6 months	30 (4.1%)	19 (2.6%)	1.58	0.90–2.78	0.1455
12 months	40 (5.5%)	23 (3.1%)	1.74	1.05–2.88	0.0386
18 months	56 (7.7%)	34 (4.6%)	1.65	1.09–2.49	0.0219
24 months	66 (9.0%)	40 (5.5%)	1.65	1.13–2.41	0.0114

Risk ratios (RR) of conversion or supplementation of an alternative class of immunomodulatory therapy (IMT) in patients with chronic uveitis treated initially with conventional IMT and compared to matched patients treated initially on biologic agents. After propensity matching, a total of 732 patients were analyzed in each cohort

## Discussion

In this large real-world study of patients treated for chronic uveitis, the impact of different IMT treatment strategies in maintaining quiescence over time was assessed. All patients in the current study had achieved an initial period of steroid-sparing control indicating that these agents were effective in inducing quiescence. However, the findings of this study highlight that the choice of maintenance therapy impacts the long-term rate of flares in these patients. Patients treated with combination therapy, with a conventional IMT and an anti-TNF-α biologic agent, experienced the fewest uveitis flares, nearly 50% less frequent than those on conventional IMT alone. This association is consistent with findings from the Bristol SYCAMORE randomized controlled trial, which demonstrated a significantly higher rate of treatment success when adalimumab was combined with methotrexate compared with methotrexate alone in patients with juvenile idiopathic arthritis-associated uveitis [15, 16].

Maintaining quiescence in chronic uveitis is critical as inflammation tends to cause cumulative damage with each recurrent episode. Even if vision partially recovers with treatment of a flare, repeated episodes tend to result in a stepwise, “saw-tooth” decline in visual function over time [3, 17]. Although the absolute rate of uveitis flares was quite low in this study, it is important to keep in mind that due to the limitations of the study design, only flares necessitating oral steroids could be captured. In practice, mild-to-moderate anterior uveitis flares can often be managed with topical prednisolone alone, whereas more severe flares, especially those with posterior segment involvement, necessitate oral corticosteroids [1, 7]. The latter type of flare is also more likely to cause lasting vision loss and thus, is of particular interest [1]. For example, patients with intermediate, posterior, or panuveitis generally have worse visual outcomes than those with isolated anterior uveitis [1]. One large study reported a median final visual acuity of approximately 20/60 in non-anterior uveitis compared to 20/30 in anterior uveitis, and a significantly higher incidence of severe vision loss in the non-anterior uveitis group [18]. Within this context, a 24-month flare rate of 3.4% on combination therapy versus 6.1% on conventional IMT may represent a meaningful reduction in cumulative risk of vision loss at the population level.

The current study also hinted a trend toward superior flare control with biologic TNF-α inhibitors compared to conventional IMTs. In the propensity-matched analysis, the risk of flare on conventional IMT was consistently 1.4 to 1.5 times that on biologic monotherapy at each time point, although these differences did not reach statistical significance. This lack of significance may be due to the modest size of the biologic agent monotherapy cohort (*n* = 732 after matching) and the substantial heterogeneity inherent to uveitis. Part of this heterogeneity was minimized by excluding patients with systemic autoimmune diseases (e.g. juvenile idiopathic arthritis, sarcoidosis, etc.) that not only bias the initial choice of IMT but also carry distinct prognoses. Nonetheless, even among cases of idiopathic uveitis, clinical behavior varies widely. For instance, indolent pars planitis has a very different flare tendency than a fulminant entity like relentless placoid chorioretinitis [19, 20]. This variability poses a challenge to any comparative analysis. Stratifying by anatomical or etiologic subtype of uveitis may allow for a more granular approach, but may introduce bias in a retrospective study and reduce statistical power. An alternative approach is a prospective study design. The ADVISE trial was a multicenter randomized trial specifically comparing adalimumab to conventional IMT as first-line steroid-sparing treatment in non-infectious intermediate, posterior, and panuveitis [12]. This trial found that patients initially treated with adalimumab achieved higher rates of tapering to ≤7.5 mg prednisone at 6 months (69% vs. 54%, *p* = 0.029) as well as higher rates of complete steroid discontinuation at 12 months (55% vs 40% *p* = 0.028) [12]. Although these findings suggest that TNF-α inhibitors may provide superior steroid-sparing effects compared to conventional IMT, the trial did not evaluate the long-term maintenance of these drugs on disease quiescence, as was reported in the current study. Furthermore, the ADVISE trial did not assess the efficacy of combination therapy, which was associated with a lower cumulative incidence of uveitis flares in the current study.

One particular advantage of combination therapy is the theoretical reduction in anti-drug antibodies (ADA). ADAs can develop in up to 50% of patients with uveitis receiving biologic monotherapy, and have been found to accelerate drug clearance resulting in reduced efficacy [21]. The combination of conventional IMT with biologics may suppress this immunogenic response and have been demonstrated to reduce the formation of ADAs in rheumatologic diseases [22]. This mechanism may partly explain the improved efficacy observed in the combination therapy cohort; however, the current study lacks the granular data needed to directly evaluate this relationship.

The difference in rate of flares between conventional IMT and biologic therapy in the current study was not found to be significantly different. However, this finding should be interpreted within the context of how these different agents are employed, as confounding by indication is possible. Conventional agents such as methotrexate and mycophenolate are often first-line therapies due to their well-established efficacy profile and lower cost [6, 7]. In contrast, biologic agents are often employed in patients with refractory disease or those with severe uveitic phenotypes, such as Behçets disease, that carry a higher risk of ocular morbidity [23]. As a result, it is possible that patients receiving biologic therapy may represent a population with a more severe or treatment-resistant uveitis, which could mask potential differences in flare rates between groups.

Although the current study did not show a significant difference in the rate of flares between conventional IMT and biologic therapy, the secondary analysis demonstrated that patients managed with conventional IMT had significantly higher rates of therapy augmentation by either switching or adding biologic agents at all time points after 1 year. Part of this pattern could reflect the efficacy differences discussed above regarding flares and control. Another factor, however, may be medication tolerability. Previous studies have noted that methotrexate can cause dose-limiting side effects, most notably hepatotoxicity, leading to discontinuation in roughly 10% of uveitis patients over the first year. Mycophenolate mofetil is generally well tolerated, but gastrointestinal intolerance still results in about 12% of patients stopping the drug within the first year [24]. By contrast, TNF-α inhibitors tend to be better tolerated; in a large real-world cohort of uveitis patients on adalimumab, only 9% discontinued therapy due to adverse effects over a median of four years [25]. Unfortunately, the design of the current study cannot distinguish whether a given therapy change was prompted by inefficacy or by side effects. Nonetheless, clinical experience suggests that in uveitis management, lack of adequate control is the dominant reason for adding a second agent rather than toxicity in most cases. Prior studies and expert surveys have noted that uveitis specialists often opt for combination systemic therapy if the response to monotherapy is suboptimal, rather than sequentially trying multiple single agents.

While the current study leverages a large multi-center dataset with robust matching to compare outcomes, it has several limitations. First, given the retrospective design, there may be residual confounding by indication. For example, patients selected for biologics might have had more severe uveitis to begin with, which could bias outcomes against the biologic group. The study attempted to minimize this by matching baseline characteristics and excluding patients with systemic inflammatory diseases that could potentially influence the decision for the initial IMT. Second, the reliance on diagnostic and pharmacy codes as proxies for clinical events is an important caveat. The study relied on EHR-based changes in prescription patterns to define corticosteroid-sparing disease control and flares due to the lack of ophthalmic examination data available on TriNetX. The SUN criteria define a flare of uveitis as a ≥ 2-step increase in inflammatory grade or new posterior segment lesions along with escalation of therapy to control inflammation [14]. The current study relied on the latter half of the definition resulting in the possibility that some flares were misclassified or missed. For example, if a patient with uveitis and asthma was prescribed a short course of oral prednisone, this could erroneously count as an ocular flare. Conversely, defining flares as initiation of ≥10 mg/day of oral prednisone likely underestimates the true rate of uveitis relapses since milder episodes managed with topical corticosteroids would not be captured. Therefore, the flares identified in this study are likely more representative of moderate-to-severe disease activity. Finally, the TriNetX platform is biased towards academic centers that participate in information sharing and thus introduces a referral bias, where the patients in this study have more severe or refractory uveitis than the average patient population. It also could mean that combination therapy is used more readily at these centers for difficult cases, which might not reflect community practice patterns.

## Conclusion

In conclusion, the current study indicates that for patients with chronic uveitis who achieve steroid-sparing control on IMT, those treated with a combination of a conventional IMT and a biologic TNF-α inhibitor have a lower risk of disease activation as compared to those on conventional IMT. Additionally, patients achieving steroid-sparing control on conventional IMT alone had higher rates of augmentation or switching to biologic therapy than those initially started on biologics. Prospective studies and clinical trials will be essential to confirm these benefits and to weigh them against the higher costs and potential risks of biologic therapy.

## Electronic supplementary material

Below is the link to the electronic supplementary material.


Supplementary Material 1


## Data Availability

The datasets generated and analyzed during this study were obtained from the TriNetX US Collaborative Network and are available from the corresponding author upon reasonable request, subject to approval by TriNetX and the institution through which the data were accessed.

## Declarations

### Meeting presentation

This work was accepted for poster presentation at the 2026 Association for Research in Vision and Ophthalmology (ARVO) Annual Meeting in Denver, Colorado, and received a National Eye Institute Travel Grant for this meeting.
