# Supplementary material for: Comparative long-term outcomes of conventional immunomodulatory therapy, biologic agents, and combination therapy in chronic uveitis
Source: J Ophthalmic Inflamm Infect. 2026 May 8;16:50. doi: 10.1186/s12348-026-00591-0 (PMC13534374; doi:10.1186/s12348-026-00591-0)
Supplement: Supplementary file 1 [file 12348_2026_591_MOESM1_ESM.docx]

| **Category** | **Disease/ Condition/ Medication** | **Code Type** | **Code** |
| --- | --- | --- | --- |
| Diagnosis | Iridocyclitis | ICD-10 | H20.X |
| Diagnosis | Posterior Cyclitis | ICD-10 | H30.2X |
| Diagnosis | Chorioretinal Inflammation | ICD-10 | H30.X |
| Diagnosis | Panuveitis | ICD-10 | H44.11 |
| Medication | Prednisone | RxNorm | 8640 |
| Medication | Prednisolone Acetate Ophthalmic Suspension 1% | RxNorm | 1376336 |
| Medication | Prednisolone Sodium Phosphate 1% | RxNorm | 314165 |
| Medication | Difluprednate 0.05% | RxNorm | 23043 |
| Medication | Methotrexate | RxNorm | 6851 |
| Medication | Mycophenolate Mofetil | RxNorm | 68149 |
| Medication | Azathioprine | RxNorm | 1256 |
| Medication | Infliximab | RxNorm | 191831 |
| Medication | Adalimumab | RxNorm | 326361 |
| Diagnosis | Crohn’s Disease | ICD-10 | K50 |
| Diagnosis | Ulcerative Colitis | ICD-10 | K51 |
| Diagnosis | Psoriasis | ICD-10 | L40 |
| Diagnosis | Systemic Lupus Erythematosus | ICD-10 | L93 |
| Diagnosis | Rheumatoid Arthritis | ICD-10 | M05-M06 |
| Diagnosis | Juvenile Idiopathic Arthritis | ICD-10 | M08 |
| Diagnosis | Ankylosing Spondylitis | ICD-10 | M45 |
| Diagnosis | Other Inflammatory Spondyloarthropathies | ICD-10 | M46 |
| Diagnosis | Nicotine Dependence | ICD-10 | F17.2X |

Supplemental Table 1: List of International Classification of Diseases, 10th Revision (ICD-10) codes and RxNorm codes utilized in the current study.
